# A randomized phase II study of S-1 monotherapy versus cisplatin with vinorelbine for completely resected stage II/IIIA non-small cell lung cancer: rationale and study protocol design for the LOGIK1702 study

**DOI:** 10.1186/s12885-021-07945-y

**Published:** 2021-03-08

**Authors:** Tomoshi Tsuchiya, Keitaro Matsumoto, Takuro Miyazaki, Ryoichiro Doi, Shoji Tokunaga, Hiroyuki Yamaguchi, Koichi Tomoshige, Hironosuke Watanabe, Takeshi Nagayasu, Kenji Sugio

**Affiliations:** 1 Department of Surgical Oncology, Nagasaki University Graduate School of Biomedical Sciences, 1-7-1 Sakamoto, Nagasaki City, 852-8501 Japan; 2 Medical Information Center, Kyushu University Hospital, Kyushu, Japan; 3 Second department of Internal medicine, Nagasaki University Hospital, Nagasaki, Japan; 4 Department of Thoracic and Breast Surgery, Oita University, Oita, Japan

**Keywords:** Adjuvant chemotherapy, S-1, Cisplatin, Feasibility study, Quality of life non-small cell lung cancer

## Abstract

**Background:**

The current standard postoperative treatment for stage II-IIIA non-small cell lung cancer (NSCLC) is a regimen of platinum doublet adjuvant chemotherapy. These regimens, which are the same as for solid NSCLC tumors, often cause severe adverse reactions in the treated patients. Therefore, an effective treatment regimen with fewer side effects is needed.

**Methods/design:**

The purpose of this study is to evaluate the effectiveness and safety of S-1 monotherapy (80 mg/m^2^ orally administrated twice daily, at day 1–14, 16 cycles) and cisplatin with vinorelbine combination therapy (cisplatin 80 mg/m^2^ at day 1,vinorelbine 25 mg/m^2^ at day 1, 8, 4 cycles) in patients with II/IIIA stage non-small-cell lung cancer who underwent a total resection. In addition, we will also evaluate the level of treatment side effects by assessing quality of life (QOL), work productivity and activity performance. The primary endpoint is a 2-year relapse free survival (RFS) and the second primary endpoints are 2-year overall survival (OS), rate of treatment completion, safety, work productivity and activity, and quality of adjusted life years (QALY). At the same time, we aim to obtain precise information required to perform future phase 3 randomized controlled trials. The study is designed to estimate the primary endpoint with accuracy determined as the width of its 95% confidence interval to be less than 20%. Recruitment started in May 2017 and is ongoing.

**Discussion:**

This study has been conceived to establish a superior regimen for completely resected NSCLC based on efficacy, safety and QOL.

**Trial registration:**

Registry number: UMIN000027435. Registered May 22, 2017.

## Background

Lung cancer remains the leading cause of cancer-related deaths worldwide. In order to prevent lung cancer recurrence, adjuvant chemotherapy in post-operative patients with stage IB-IIIA non-small cell lung cancer (NSCLC) has been established as the standard treatment [1–3]. The standard regimen for adjuvant chemotherapy is intravenous administration of a platinum doublet [3]. However, the representative regimen of cisplatin (CDDP) with vinorelbine (VNR) causes grade three or four adverse reactions including severe leukopenia, neutropenia, anemia, aspartate aminotransferase (AST) elevation, nausea, vomiting, constipation, asthenia, injection site reaction, and prolonged peripheral neuropathy in more than 70% of patients. Renal dysfunction often occurs and sometimes remains lifelong. Given the severe adverse reactions to this standard adjuvant chemotherapy and the fact that there are few remnant malignant cells in the postoperative patients, milder treatment options have been considered.

A recent study of the 5-year survival in a randomized phase III study of cisplatin with pemetrexed versus cisplatin with vinorelbine for completely resected stage II-IIIA non-squamous NSCLC (the JIPANG study) was reported (ASCO 2019 8501). The JIPANG study failed to show the superiority of pemetrexed plus cisplatin over vinorelbine plus cisplatin and the cost of pemetrexed is high. However, the regimen will be considered as an option for adjuvant chemotherapy for stage II-IIIA non-squamous NSCLC since the effect is comparable and the patients treated with cisplatin and pemetrexed had a higher completion rate with low adverse reactions compared to the patients treated with cisplatin and vinorelbine. Another recent approach is the use of immune checkpoint inhibitors (ICI) for postoperative patients. Several clinical trials of ICI with/without chemotherapy are currently underway in adjuvant setting [4]. The trials are expected to be completed during 2024 or later. Thus the efficacy and the adverse reactions of ICI in adjuvant therapy remain unclear.

S-1 might be another candidate for mild adjuvant chemotherapy for NSCLC. The oral fluoropyrimidine derivative which consists of tegafur and two modulators, 5-chloro-2,4-dihydroxypyridine (CDHP) and potassium oxonate (Oxo), enhances 5-FU efficacy by inhibiting degradation of 5-FU and reduces the GI toxicity caused by 5-FU [5]. The significant effects with low adverse reactions of S-1 monotherapy in an adjuvant treatment have been proven in randomized phase III trials in digestive cancers including gastric, pancreatic and colon adenocarcinomas [6–8]. Accordingly, S-1 based adjuvant chemotherapy has been used as an option of standard treatment in intestinal cancers. Further, S-1 also has efficacy against squamous cell carcinomas including head and neck, oral, esophagus and thymus [9–11], suggesting S-1 is effective on both squamous carcinomas and adenocarcinomas.

Feasibility studies of S-1 based adjuvant chemotherapy for patients with stage IB to IIIA NSCLC have also been conducted [12, 13] . The results indicate that the completion rates of planned one-year treatment courses were comparable to platinum doublet (50–72%) and the grade 3 adverse reactions were very low (4–20%) with no grade 4 adverse reactions. The grade 3/4 neutropenia was extremely low (0–6.7%), compared to the meta-analysis of cisplatin with vinorelbine adjuvant chemotherapy of 80% [14]. The 5-year overall and relapse free survival rates of the S-1 treated postoperative patients with stage IB-IIIA NSCLC were 72.5 and 67.5%, which are obviously better than those of previously reported studies including platinum doublet based adjuvant chemotherapies [12]. The randomized phase II study of adjuvant chemotherapy with S-1 and cisplatin with S-1 revealed that the survival rates of both groups overlap in patients with completely resected stage II–IIIA NSCLC [15], indicating that S-1 monotherapy has the potential for replacing the standard regimen of platinum doublet by virtue of its efficacy and safety.

The low incidence of adverse reaction directly correlates with the quality of life of the treated patients. Because of mild adverse reactions, S-1 can be administrated for outpatients without hospitalization and treated patients can continue daily life and working, which will maintain the social productivity of the treated individuals. The evaluation of quality of life (QOL) and work productivity of postoperative patients shows the advantage of S-1 chemotherapy versus standard platinum doublet adjuvant chemotherapy. Taken together, these findings have enabled us to design the current phase II study to confirm the beneficial effects of S-1 in adjuvant chemotherapy. In this article, we describe the protocol (version 2; January 14, 2020) for this study. The results of this study are expected to provide basic information for raising the precision of performance for a randomized phase III study.

## Methods/design

### Study design and treatment

**Fig. 1:**
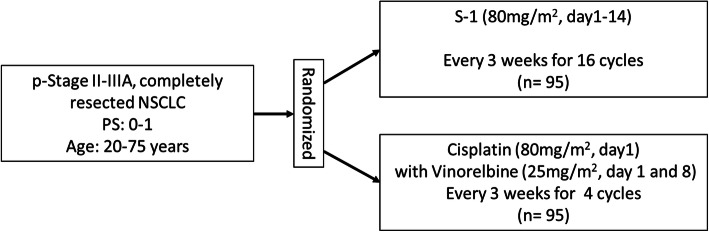
Study Design of LOGIK1702 Study, A Randomized Phase II Study of S-1 and Cisplatin with Vinorelbine for Completely Resected Stage II/IIIA NSCLC

**Fig. 2:**
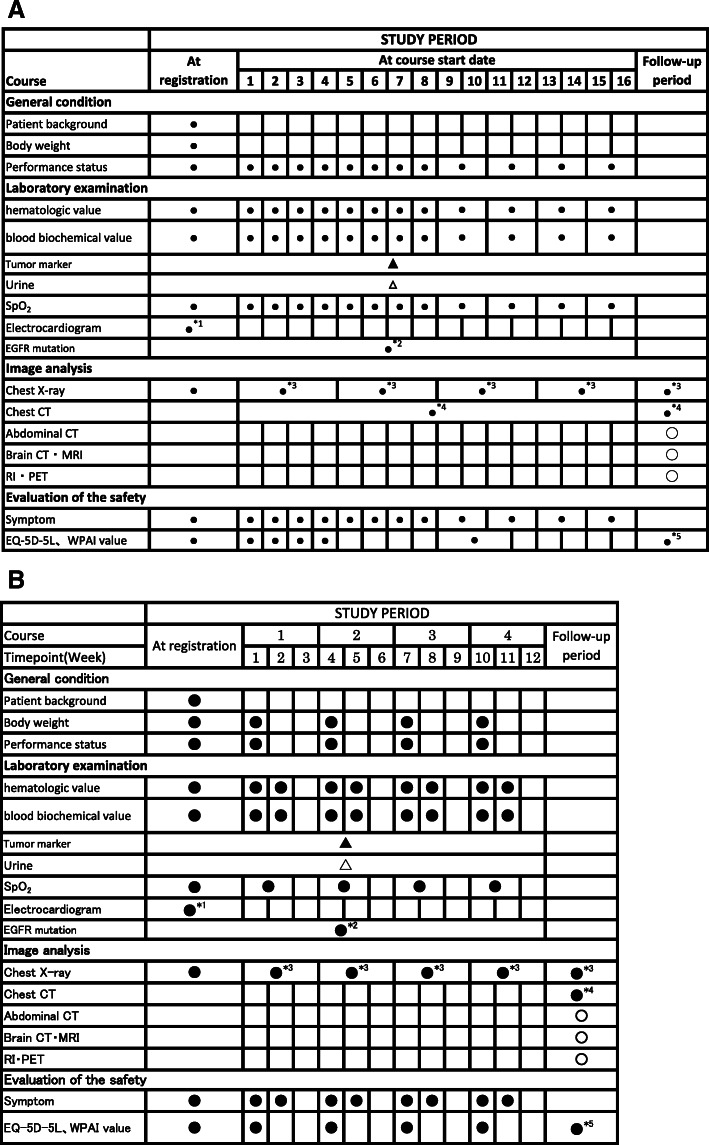
Treatment schedule and outcome measures of arm A (2A) and arm B (2B). ●; Measurements, ▲; Measurable facilities (Once a month), △; optional. *1; Measurements at every 90 (±30) days until 3 years from the start of the treatment. Every 180 (±60) days thereafter.*2; Measurements at every 180 (±60) days until 3 years from the start of the treatment. Every 365 (±90) days thereafter. *3: Measurements at 365 (±60) days and 545(±60) days. *CT Computed tomography, MRI Magnetic resonance imaging, RI Radio isotope, PET Positron emission tomography, WPAI Work productivity and activity impairment questionnaire*

The present study is a multicenter, phase II, randomized, open-labelled, parallel-group comparison study of the efficacy and safety of S-1 compared with cisplatin plus vinorelbine for adjuvant therapy in patients with NSCLC. The study design is summarized in Fig. 1. Patients are randomized in a 1:1 ratio to the experimental arm (1 year of S-1 administration) or control arm (four cycles of cisplatin with vinorelbine administration), stratified according to sex (female vs. male), age (< 70 years vs. 70 years), pathologic stage (II vs. IIIA) and pathology (squamous cell carcinoma vs. non- squamous cell carcinoma). The primary endpoint is 2-year relapse-free survival (RFS). The secondary endpoints are 2-year overall survival (OS), rate of treatment completion, safety, work productivity and activity, and quality of adjusted life years (QALY). QALY is calculated by the time points of QOL converted from the questionnaire method using Euro QoL 5 dimensions (EQ-5D-5L) [16] and conversion chart. The EQ-5D-5L and work productivity and activity impairment (WPAI) are surveyed by questionnaires for one and a half years from the start of adjuvant chemotherapy (Fig. 2).

The study is being conducted at 30 institutions in the Lung Cancer Group in Kyusyu (LOGIK) in Japan. The study is registered with the University Hospital Medical Information Network Clinical Trials Registry (www.umin.ac.jp/ctr/) under registration number UMIN000027435. We conduct the study in accordance with the principles of the Declaration of Helsinki. The central ethics committee approved the study.

All eligible patients will be selected and approached on the basis of information derived from the electronic health records of these 30 institutions (academic hospitals) according to the inclusion and exclusion criteria. Participants will be provided with an explanation about the study by their treating physicians, and they will be asked to voluntarily sign an informed consent before participation. If the patient’s consent is obtained, a clinical trial physician will perform the observation/examination based on the description in Fig. 2a and b.

### Eligibility criteria

The main patient inclusion and exclusion criteria are detailed in Table 1.

**Table 1:** Main Inclusion and Exclusion Criteria

Inclusion Criteria	
1) Patients providing the written informed consent	
2) Patients with non-small-cell lung cancer histopathologically confirmed	
3) Patients with total resection in pathological clinical stage II - IIIA with more than one lobe excision was carried out	
4) Patients with lymphadenectomy more than ND2a-1 or selective lymphadenectomy	
The selection method of the mediastinal lymph node dissected obeys the following criteria	
●Right upper lobe lung cancer (LC); No.2R,4R	
●Left upper lobe segmentum superius LC; No. 4 L-6	
●Right middle lobe LC;	No.2R,4R,7
●Left upper lobe lingual LC; No.4 L-7	
●Right lower lobe LC; No.7–9	
●Left lower lobe LC; No.7–9	
5) Patients without pre-treatment (radiation, chemotherapy) other than surgical treatment	
6) Patients who passed more than 28 days and less than 56 days after the operation at enrollment	
7) Patient is at least 20 years and less than 75 years of age (at enrollment date).	
8) Patients capable of treatment with oral medicine.	
9) Eastern Cooperative Oncology Group (ECOG) performance status of 0–1	
10) Patients having sufficient bone marrow, liver and renal function tolerable to chemotherapy	
Exclusion Criteria	
1) Patients with active double cancer (synchronous double cancer and asynchronous double cancer within 5 years of progression-free period)	
2) Patients with anamnesis of drug-induced hypersensitivity.	
3) Patients with severe postoperative complications (such as postoperative infection, rapture suture).	
4) Patients with severe complications (such as diarrhoea, intestinal paralysis, ileus, uncontrollable diabetes mellitus, heart failure, kidney failure, liver failure).	
5) Patients with interstitial pattern recognized as apparent interstitial pneumonia in chest CT.	
6) Patients with active infection.	
7) Female patients pregnant or possibly pregnant (will), or nursing.	
8) Patients under treatment with a type of fluorinated pyrimidine antineoplastic.	
9) Patients under treatment of flucytosine.	

### Treatment

Arm A (S-1 monotherapy) is an adjuvant therapy of S-1 for 16 courses (1 year). S-1 80 mg/m^2^ is orally administered twice daily (after breakfast and dinner) from after breakfast at day 1 to after dinner at day 14 (or from after dinner at day1 to after breakfast at day 15) as 21 days of 1 course.

Arm B (Cisplatin with vinorelbine combination therapy) is an adjuvant therapy of cisplatin with vinorelbine for 4 courses. Cisplatin 80 mg/m^2^ at day1 and vinorelbine 25 mg/m^2^ at day 1, 8 are administered as 21 days of 1 course for 4 courses.

### Randomization

After the acquisition of written informed consent and the completion of baseline measurements, the enrolled participants are registered and assigned treatment by the registration center. Participants will be randomly allocated at a ratio of 1:1 (S1 versus Cisplatin with vinorelbine). Randomization will be performed using the block randomization method with randomly varying block length utilizing SAS version 9.4 software (SAS Institute, Cary, NC, USA).

### Statistical design and sample size considerations

This aim of this study is to estimate the 2 year relapse-free survival rate (RFS) for each study arm with sufficient clinical precision. The statistical comparison of the difference in RFS between the two arms is not a primary aim. We designed the sample size to estimate the 2-year-RFS with the accuracy determined to be the width of the 95% confidence interval for the 2 year-RFS to be less than 20%. The 2-year RFS was expected to be 65%. Assuming an estimate at the 95% confidence interval for the 2 year-RFS with the Wilson’s method, 84 patients are necessary for each arm. Estimating around 10% drop outs, we set the total number of patients to be 190. The above sample size was set to assure the statistical power to quantitatively compare the primary point between the arms. The primary goal of this study is to evaluate the clinical significance of the S1 monotherapy by integrating the observed data of the RFSs, QOL and adverse events of the two arms.

Relapse-free survival was defined as the time from the date of the start of treatment to the date of disease progression or death (whichever occurs first) or the date of last contact. Overall survival was defined as the time from the date of the start of treatment to the date of death or last contact. The Kaplan-Meier method will be used to estimate the time-to-event functions of relapse-free survival and overall survival. The log-rank test will be conducted as a reference without setting the significance level.

## Discussion

Since 2004, cisplatin with vinorelbine has been the preferred treatment for patients who have undergone complete resection of stage II to IIIA NSCLC and the recommended regimen has not been changed in more than 16 years. Because the adverse reactions are severe in the cisplatin with vinorelbine regime, the Lung Cancer Group in Kyusyu (LOGIK) is conducting the LOGIK1702 study in order to compare the efficacy of milder chemotherapy using S-1 compared to the standard regimen of cisplatin with vinorelbine. S-1 chemotherapy has been successfully used for other cancers and is recommended for intestinal adjuvant chemotherapy. In previous studies using cisplatin doublet based adjuvant chemotherapies most of the relapses occurred within 2-years following the operation and the survival curve came to a plateau thereafter [1–3, 15]. In order to estimate the efficacy of the two regimens, the primary endpoint of this study is also configured as 2-year RFS. The other aspect of this study is to evaluate work productivity and activity, and quality of adjusted life years (QALY) by using the questionnaire of the EQ-5D-5L, which was configured as a secondary endpoint. The EQ-5D-5L measures five aspects of the patient’s life: mobility, self-care, usual activities, pain/discomfort and anxiety/depression. Accordingly, the patients’ QALY will be expressed numerically and evaluated objectively. Since better QOL and work productivity are also desired even for postoperative patients, this analysis will evaluate another aspect of the adjuvant chemotherapy in addition to prognosis. Quality of life is important as well as the longevity prognosis. To the best of our knowledge, this is the first prospective study which evaluates the QALY and work productivity in postoperative adjuvant chemotherapy.

The limitation of the present study is that this randomized study is not aimed at evaluating statistical comparison of the difference in prognoses between the two arms. Rather, this study is aimed at providing basic information for improving the performance evaluation of a future randomized phase III study. The LOGIK1702 study will give us new information on the optimal chemotherapy regimen for completely resected NSCLC.

## Data Availability

Only the statistician who designed this trial will have access to the personal data of participants and to the final data set. The datasets used and/or analysed during the current study will be available from the corresponding author on reasonable request.

## Abbreviations

NSCLC: Non-small cell lung cancer
QOL: Quality of life
RFS: Relapse free survival
OS: Overall survival
QALY: Quality of adjusted life years
CDDP: Cisplatin
VNR: Vinorelbine
AST: Aspartate aminotransferase
CDHP: 5-chloro-2,4-dihydroxypyridine
Oxo: Potassium oxonate
EQ-5D-5L: Euro QoL 5 dimensions
WPAI: Work productivity and activity impairment
